# Opportunities and counterintuitive challenges for decentralized clinical trials to broaden participant inclusion

**DOI:** 10.1038/s41746-022-00603-y

**Published:** 2022-05-05

**Authors:** Noah Goodson, Paul Wicks, Jayne Morgan, Leen Hashem, Sinéad Callinan, John Reites

**Affiliations:** 1 THREAD Research, 155 El Camino Real, Tustin, CA 92780 USA; 2 Wicks Digital Health, Advantage House, Lichfield, Staffordshire WS13 6AQ UK; 3 Piedmont Health, 2727 Paces Ferry Road SE Building 2, Suite 1100, Atlanta, GA 30339 USA

**Keywords:** Clinical trial design, Clinical trials

## Abstract

Traditional clinical trials have often failed to recruit representative participant populations. Just 5% of eligible patients participate in clinical research. Participants, particularly those from minority groups, cite geographical constraints, mistrust, miscommunication, and discrimination as barriers. Here, an intersectional view of inclusion in clinical trials provides significant insights into the complex and counterintuitive challenges of trial design and participant recruitment. The US FDA have recently proposed that decentralized clinical trials (DCTs) might reduce barriers and appeal to a wider range of participants by reducing the costs and commitments required for patients to participate. While common sense and early evidence suggests that allowing participants to take part in trials at or near home has advantages in terms of convenience, travel, and perhaps even infection control, it remains to be seen if DCT approaches will yield significant improvements on participant inclusivity. Some digital studies aiming to be more inclusive on a single element of inclusion, such as race, have experienced unintended consequences in other elements, like education or gender. Implementing DCTs presents new challenges including the digital divide, the exclusion of certain tests and procedures, complexities of at-home medication delivery, and the need to build new infrastructure. We present a range of challenges and opportunities for researchers to adopt and adapt DCT approaches to create reliable evidence that applies to all of us.

## Introduction

Evidence-based medicine should draw upon the results of inclusive and representative trials in order to be robust^1^. However, it is clear that women, older adults, Black, Indigenous, and other People of Color (BIPOC), as well as those with comorbidities are frequently under-represented in trials^2^. For example, Black participants are poorly represented in key areas like oncology and cardiovascular trials, but over-represented in psychiatry trials^3^. These disparities are problematic on many levels, including the generalizability of results and social justice matters^4^. Underrepresentation is also a practical challenge for health systems, given that disease profiles vary significantly across marginalized groups^5^.

Catalyzed by the impact of the COVID-19 pandemic^6^, medical researchers^7^ and the US FDA have proposed that DCT approaches “may ease the burden of participating in a clinical trial and potentially improve recruitment and retention of diverse participants”^8^. Unlike a traditional site-based design, which requires participants to visit study sites regularly, DCTs combine software, technology, and healthcare professional visits to allow some or all trial visits to take place away from the trial site—for example, at the participant’s residence^9^. However, we argue that simply using more technology will not suffice to improve inclusion. In this Perspective, we outline the potential benefits for using DCTs to improve participant inclusion in clinical trials. We also highlight the challenges arising and make recommendations for how the researchers conducting them could do more to address this topic.

### Inclusion and diversity in clinical trials

Despite the need to develop effective new treatments, less than 5% of eligible patients participate in clinical research^10^, a situation even more pronounced for racial and ethnic minorities^11^. Up to 20% of clinical trials are either terminated early for failing to meet recruitment targets or are completed while failing to meet the original target^12^. The pressure to successfully recruit enough participants of any kind might make the demand to consider inclusivity in trial design seem onerous. However, we propose that a trial designed with inclusivity in mind from study initiation will be more successful on all fronts.

Any potential clinical trial participant may be dramatically different in culture, experience, and values from the healthcare professional that designs a study^13^ or presents a potential trial^14^. The barriers and opportunities for trial participation are complex, as demonstrated by a recent “review of reviews”, which identified 881 relevant factors grouped into 20 themes^15^. Many barriers to participation are not thought to be specific to a given trial but rather relate to participants’ attitudinal barriers, low levels of research awareness, and mistrust of institutions, as well as structural health inequalities and racism, for example^16^. Thus, clinicians and patients are likely to differ in a range of attributes that collectively alter their views of what is important and how those views translate to decision making across the health spectrum^14^.

Studies have historically gauged inclusivity and representativeness in a unidimensional fashion, reporting simple “percentages” of identifiable demographics. These inclusivity categories commonly encompass age, sex, gender identity, race, ethnicity, sexual orientation, language use (or native tongue), reproductive status, pregnancy status, breast feeding status, socioeconomic status, living arrangements, disability, comorbidities, education, health literacy, obesity, geography, health insurance, digital divide, rurality, substance use, occupation, social capital, and vulnerable populations. However, it has been found that strategies that attempt to address any one of these categories can cause imbalances in others.

For example, campaigns using online methods to recruit more racially diverse participants result in a disproportionate increase in the enrollment of females, the highly educated, and the wealthy^17^. This may seem like an unsolvable challenge when each of these population features seem to overlap. However, the crux of the issue for many participants is characterized by their *intersectionality*. In other words, the power imbalance of social inequalities do not necessarily privilege any *one* of these factors in isolation (e.g., age, gender, race), but rather each individual participant sits at the intersection of many of these categories with their own degree of opportunity and oppression^13^. Intersectionality as a concept arose from Black critical thinkers and activists who identified that the experience of an individual who is over 65 years old, Black, and female is more pronounced than the experience of an individual who is *either* over 65, or Black, or female^13^—the whole is greater than the sum of its parts.

When viewed through an intersectional framework, the challenges of recruiting diverse clinical trial populations become clearer. The frequent failures to adequately recruit representative populations are not simply a function of poorly phrased advertising or the incorrect selection of study sites, although these factors may also be meaningful. Instead, poor inclusion, as a systemic problem, may be impacted by the way clinical trials are conducted across the healthcare spectrum. The combined institutional and societal forces create an intersectional suite of barriers for people whose experience is more than the sum of its parts.

### Opportunities for DCT approaches to improve inclusivity

The most obvious difference between DCTs and traditional site-based designs is geography, which affects multiple and intersectional facets of inclusion. Traditionally designed clinical trials typically use sites based at urban academic-affiliated specialist centers. Decentralized approaches often rely on technology, like mobile phone applications, to engage with patients, but may also utilize more dispersed local clinics or home health providers to “decentralize” some, or all data collected in a trial. Many potential participants, even those with conditions serious enough to ensure continued research engagement, are unwilling to travel for many hours for a traditional study^14^, and may withdraw from a trial if subjected to long waits at trial sites^18^. Groups typically excluded from clinical trials are disproportionately affected by geographical constraints. For example, females often shoulder more childcare and family caring responsibilities than males, suffer a payment gap that reduces their economic power, and feel less able to take time off work than their male counterparts^19^. This situation is compounded for women who are BIPOC, LGBTQ + , or disabled.

Decentralized clinical trials remove many of the geographical constraints faced by trial participants^15^, with subsequent potential benefits for minimizing participant investment in time, expenses, reliance on caregivers, or having to arrange childcare^20^. During the initial COVID-19 pandemic, several studies were conducted using fully decentralized approaches, removing all in-person interactions. One such study recruited 821 participants, primarily through social media, to test hydroxychloroquine as prophylaxis against COVID-19 infection after potential exposure, using online surveys for follow-up^21^. The North-America based study quickly recruited a representative sample on sex (51.6% female participants) from a wider geography than is typical for traditional trials. White participants accounted for 61.7% of the sample (compared to an average of 80-84% white participants in other US-based trials^22–24^. In a second, fully decentralized study in the United States studying the impact of fluvoxamine on the progression of COVID-19 symptoms, 25% of participants identified as Black^25^, far more than standard recruitment rates of around 4% in the United States^22–24^. However, limited sample size and regional recruitment limitations suggest to these authors caution around generalizing results from this study.

As trials are increasingly globalized, the impact of DCT approaches may be particularly relevant outside the US, as regions like South America, Eastern Europe, Eurasia, and Pacific countries have even lower representation of female trial participants^3^. People with disabilities affecting movement are especially likely to benefit from DCT approaches, such as participants with Parkinson’s disease who stand to save two hours in travel time per visit and 88 miles (141.6 km) in travel distance to and from study sites^26^. In the case of rare diseases, such as amyotrophic lateral sclerosis, most patients (71%) live more than 25 miles away from trial sites, with more challenging geographic distances for minority ethnic groups^27^.

Another common element of DCT approaches is electronic informed consent (eConsent), a feature that may be supported by a series of video vignettes, which explain the study in an engaging format. Patients interact with eConsent solutions at their own pace, as opposed to potentially feeling rushed to read and understand lengthy paper documents in the doctor’s office. While it might be assumed that technological elements would only benefit younger, more tech-savvy participants, a trial enrolling 7904 participants found that relative to sites using traditional consent methods, sites with video consent capabilities recruited faster and enrolled more patients who were non-White, older than 75 years old, and who had lower levels of education^28^.

An additional element of DCTs is the use of telehealth or “virtual visits”. Virtual visits involve the use of a smartphone, tablet, or computer to conduct a healthcare engagement or clinical assessment via video and audio. Even before the COVID-19 pandemic, a meta-analysis showed significant patient and caregiver satisfaction across four core telehealth metrics (system experience, information sharing, consumer focus, and overall satisfaction) especially for those in rural and remote communities^29^.

Beyond age, sex, and race, there are also significant disparities in where trial participants live. In a systematic review comparing recruitment features between DCTs and traditional designs, researchers found that participants in DCTs were enrolled from an average of 40 US states in comparison to traditional clinical trials, who were enrolled from an average of just a single state^30^. DCTs in the review also recruited their target samples significantly faster (mean 4.0 months vs. 15.9 months)^30^. Such findings have also been replicated outside the US, such as in a Swiss study of low back pain where researchers found that DCT approaches led to trial enrollment that was three times faster and five times more geographically representative than conventional approaches^31^.

### Challenges for DCT approaches to improve inclusivity

DCTs may well make trial participation broadly accessible, less burdensome, and more engaging. However, some of the most significant impediments to trial participation for some minority subgroups are likely more related to structural racism than mere inconvenience^32^. It is unlikely that technology will overcome such barriers alone. Any improvements in inclusivity propelled by DCTs will also have to overcome broader industry trends that consistently make trial participation more difficult for all participants. For example, a growing number of innovative drugs over the past 4 decades have been orphan drugs, particularly in cancer trials that have restrictive inclusion/exclusion criteria^33^, the number of which has doubled in the past 10 years^34^. The number of procedures that participants must undergo during trials has also been climbing steadily since 2000^35^ and every one of these additional elements increases the perceived burden of a trial, with the potential to disproportionately affect those who are already under-represented^34^. Indeed, when viewed through a lens of intersectional inclusion, increased trial burden will almost always disproportionately impact the ability of individuals to enroll, apart from the participation of a privileged few. Similarly, the financial and economic burden of the 2008 financial crisis, climate change, and COVID-19 heavily impacts these same groups^8,36^. Against such long-term trends, we may need every DCT innovation on offer in order just to maintain the minimal status quo.

Yet without a research agenda in place to systematically apply tools like Studies Within A Trial^37^ or the INCLUDE ethnicity framework^38^, researchers conducting DCTs may miss the opportunity to share outcomes and lessons learned in broadening participant inclusion. As previously noted, improvement in one dimension may have unintended consequences on other dimensions.

Recruitment to the decentralized hydroxychloroquine study highlighted earlier was not uniform, with Asian participants over-represented and Black participants under-represented compared to the US population^21^. The contrast is even more disproportionate when compared to the impact of COVID-19 on Black and Latino communities^25^. The authors also identified that their study participants were younger, and therefore potentially healthier, than those most affected by COVID-19^21^.

Prior to COVID-19, Watson et al.^17^ designed a randomized trial for a smoking cessation intervention in the US that sought to achieve 25% representation of racial/ethnic minorities (i.e., those who did not identify as non-Hispanic Caucasians). The authors compared traditional, web-based, and online survey panel methods for recruitment. While they achieved their inclusion goals on the domain of race and ethnicity, 79% of their sample was female, perhaps due to their reliance on Internet advertising and social media, which tends to skew female. However, males smoke more than females in the US^39^. Because the authors collected data on other key demographics, they also identified other unintended biases. Their sample was also highly educated; only 28% had a high-school education or less compared with the US census-recorded attainment of 39%. Those with the lowest levels of education have smoking rates between 2-5x higher than those with a higher degree of education, meaning that the study may not address those with the highest unmet need^39^.

In another example, the PRIDE study was developed on the THREAD Research platform in collaboration with Stanford and UCSF to ensure sexual and gender minority people, who are traditionally under-represented in research, would have a secure online system for research participation to redress this imbalance^40^. In addition to the technology platform, this approach also consisted of engaging a national network of sexual and gender minority serving organizations, professional advocacy organizations, a participant advisory committee, and dedicated ambassadors to incorporate the influence of their peers. Similarly to the Watson et al. study, the PRIDE succeeded in engaging a relatively large proportion of gender minority participants (*N* = 3814 representing 32.8% of the sample relative to 0.6% national estimated rates). However, a number of other minority groups were under-represented in the sample. For example, only half the level of Hispanic ethnicity participants (8.4%) were enrolled relative to the US population (16.3%) and 65% of participants were educated to a 4-year college degree level or higher compared to 32.2% of the US adult population^40^. Ultimately, the results of such studies suggest that success in one aspect of inclusion may result in meaningful biases in other areas.

The most significant barrier to DCT adoption may well be the “digital divide.” Around 20% of the US population has access to neither broadband internet nor a smartphone and this rate is even poorer for those who are older, less educated, less wealthy, living in rural areas, or from a minority ethnic group^41^. Furthermore, procedures such as certain lab tests or surgical procedures are not amenable to remote measurement^42^, and the delivery of experimental medicines by mail is a more complex endeavor than simply dispensing through a hospital pharmacy^43^. Finally, there are fundamental concerns that must be addressed with DCT approaches including patient engagement^44^, researcher training^42^, the development and validation of new digital biomarkers^42^, reshaping existing clinical trial infrastructure to be more amenable to DCTs, and the widespread adoption and harmonization of clinical data standards^45^. Thus, while impactful and critical, decentralized clinical trial elements do not de facto solve inclusivity challenges in clinical research.

### Improving inclusion and diversity through decentralized clinical trials

As practitioners experienced in conducting over 100 clinical trials including traditional designs, fully decentralized clinical trials, and hybrid designs, we have identified several opportunities where DCTs might expand inclusivity in in the trial operations and closeout (Tables 1 and 2) phases. There are many strategies ranging from digital recruitment and screening to post-study follow-up that may provide meaningful value to communities who have been historically marginalized from the clinical research process. However, we have found that meeting the needs of the community by (1) decreasing study visit duration when possible and (2) decentralizing the study visits for more convenience are two of the most holistically advantageous strategies to increasing inclusion. This can be achieved through many mechanisms, including but not limited to completing some of the scheduled visits remotely, completing some visits virtually via telehealth rather than in person, shortening time on site by completing electronic Clinical Outcomes Assessments in a mobile application, or decreasing travel time by having samples like blood collected from a local facility.

**Table 1:** Opportunities for DCT to enhance inclusivity in the operational phases of a trial.

Operational phase	Current challenges	DCT-enabled opportunities
Recruitment	Often rely on specialist academic sites with little marketing knowledge	Involve diverse groups in recruitment strategy, use digital channels and online communities, make DCT a searchable domain in trial registries
Screening	Limited ability to screen participants in their native language or gain feedback on reasons for non-participation	Digital recruitment can lead to multilingual prescreening with continuous feedback on non-enrollment to evolve strategy
Consent	Traditional consent methods confusing for those with low health literacy, non-native language speakers. Paper consenting is inefficient, often perceived by participants as more focused on legal protection then informed process	Offer multiple approaches to ensure understanding through electronic consent process including video consenting, quizzes, and always available digital copy of information (some regions may still require ink signature)
Investigational Medicinal Product Provisioning	May require extra site visits, may not track consumption well	Augment delivery with DCT medication adherence solutions, e.g., reminders, photos, videos, smart packaging
Scheduling and reimbursement	Complex for site staff to schedule, significant burden on patients. Reimbursement paperwork, patients often left out of pocket – onerous to poorest participants	Expand trial services that can be digitally scheduled centrally, e.g., imaging, blood draws. At-home visits or organized transport minimizes costs incurred
Outcomes—Labs and objective tests	Only available in weekday working hours often at central clinics – harder for caregivers, single parents, shift workers	At-home self-collection kits increasingly familiar due to COVID-19, home healthcare visits, collect samples through local labs or pharmacies
Outcomes—Patient reported outcomes	Long, repetitive questionnaires generate a lot of paper, error-prone completion and scoring	Electronic patient report outcomes, surveys, and electronic diaries much easier to complete for many participants on mobile device, provide more secure and validated data, can be completed closer to clinically relevant temporal windows
Outcomes—Devices and objective sensors	Lack of technical expertise at sites incurs burden to set up and support devices	Digital biomarkers may be less sensitive to cultural biases, e.g., health literacy but must ensure they work across population
Outcomes—Clinician and performance reported outcomes	Busy schedules at sites or disruptions through events like pandemic may leave missing or incomplete data	Many clinician reported outcomes and standard of care practices can be completed through telehealth or home health visits - reduced burden on participants and clinicians
Side effects and safety	Retrospective participant recall of potential side effects often unreliable, reporting burdensome on sites	DCT potential to capture data in real time or closer to event experience through variety of mechanisms - increased ability for real time safety monitoring
Engagement	Traditional designs leave engagement up to study sites—may have highly variable results and inconsistent communication	Build in continuous participant feedback, provide mobile phone engagement opportunities through reminders, report modifications made in response to suggestions
Integrations	Multiple and disparate systems are used to capture trial data	Ease technology burden by providing integrated solutions and automations

**Table 2:** Opportunities for DCT to enhance inclusivity in the closeout phases of a trial.

Study closeout	Current challenges	DCT-enabled opportunities
Return of results	Due to confidentiality and site visit timing, participants may hear results first in the media or disinformation may spread online	Normalize investment in communicating study results to participants, not just to clinician, payer, or investor audiences - particularly important for traditionally marginalized communities to understand outcomes from their contribution
Post-Trial Activities	Once trial has concluded, no systematic methods for maintaining relationship with participants	Develop post-trial communication strategies to maintain relationships with communities and offer further opportunities to participate

## Conclusion

Addressing inclusivity will require a concerted and sustained effort from multiple stakeholders. Without a major paradigm shift, the drive to address trial inclusivity may take decades, or even generations. For instance, efforts to increase the number of female clinical trial participants by the US FDA started in the 1990’s but has only recently approached parity, climbing from <20% to 45%^46^. Efforts to address ethnic and racial diversity are much more recent; it has been less than 10 years since the US FDA mandated reporting of demographic subgroups reported in clinical trials^47^. An analysis of 204 oncology trials between 2008 and 2018 found one in three trials didn’t even record participant race, with no evidence of improvement from the first half of the decade to the second half^48^. Collaborations such as the Clinical Trials Transformation Initiative and the Decentralized Trials and Research Alliance provide mechanisms to lead a global research agenda that serves the needs of patients, trialists, regulators, and technology providers alike. Many collaborations similar to these will be critical to understanding the sometimes counterintuitive outcomes of decentralized approaches on participant recruitment and generate a suite of best-practices for researchers conducting DCTs. Without systemic changes in the way trials are conducted it is unlikely that current recruitment models will be able to keep up with poor enrollment numbers, much less drive social parity and equity in the way novel therapies are tested.

### Reporting summary

Further information on research design is available in the Nature Research Reporting Summary linked to this article.

## Supplementary information

Reporting Summary

## Data Availability

Data sharing is not applicable to this article as no new data were created or analyzed in this study.
